# Prognostic value of the echocardiographic-derived calcium index in coronary artery disease

**DOI:** 10.1186/cc10798

**Published:** 2012-03-20

**Authors:** J Jimenez, J Iribarren, J Lacalzada, A De La Rosa, R Juárez, A Barragán, J Bonilla, G Blanco, R Pérez, M Brouard, I Laynez

**Affiliations:** 1 Hospital Universitario de Canarias, La Laguna, Spain

## Introduction

Calcification of different cardiac structures is associated with atherosclerotic risk factors. The aim of this study is to determine whether the echocardiography-derived calcium index (ECI) assessed by transthoracic echocardiography (TTE) predicts cardiovascular events, besides determining the coronary artery calcium score (CACS), the presence of obstructive coronary artery disease (CAD) and the composition of plaques, all of which determined by multidetector computed tomography (MDCT).

## Methods

We carried out a prospective study of 82 consecutive patients, with an intermediate likelihood for CAD, who were evaluated by noninvasive coronariography by MDCT. ECI was blindly assessed by TTE. A 36-month follow-up was conducted to detect cardiovascular events.

## Results

The area under the ROC curve (AUC) of the Agatston score scale as a predictor of significant obstruction identified by MDCT was 0.80 (95% CI: 0.68 to 0.91); *P *< 0.001. The optimal cut-off was 239. Agatston score ≥239 has a sensitivity (Se) of 60.6% (95% CI: 0.42 to 0.77), specificity (Sp) of 97.8% (95% CI: 0.88 to 0.99), positive predictive value (PPV) of 95.2% and negative predictive value (NPV) of 77.2%. The AUC of ECI to predict an optimal cut-off value for Agatston score was 0.90 (95% CI: 0.83 to 0.96); *P *< 0.001. ECI ≥7 had a Se of 59.1% (95% CI: 0.36 to 0.79), a Sp of 93.3% (95% CI: 0.83 to 0.98), PPV of 76.5% and NPV of 86.2%. There was a significant linear trend of ECI, and ECI ≥7 has in MDCT a greater presence of both severe calcified wall and obstructive CAD, number of affected vessels, and mixed/calcified plaques (all *P *< 0.001). There were 23 coronary ischemic events. The AUC of ECI as a predictor of adverse cardiac events post MDCT was 0.92 (95% CI: 0.852 to 0.987); *P *< 0.001. ECI ≥7 had a Se of 77.3% (95% CI: 54.6 to 92.2), a Sp of 90% (95% CI: 79.5 to 96.2), PPV of 73.9% and NPV of 91.5%. The Kaplan-Meier survival analyses show a statistically significant difference between patients with VCSI ≥7 or not regarding an ischemic event (χ^2^: 52, *P *< 0.001). This accumulation of risk occurs mainly in the first 2 years after the determination of ECI.

## Conclusion

ECI ≥7 determines a poor CAD prognosis of coronary ischemic events. Furthermore, ECI ≥7 may serve as a marker of the content of wall calcium, obstruction level and composition of the plaques. ECI seems to provide prognostic information as well as providing information about the characteristics of the plaque of atheroma.

